# Proximity-dependent biotinylation technologies for mapping RNA-protein interactions in live cells

**DOI:** 10.3389/fmolb.2022.1062448

**Published:** 2022-11-14

**Authors:** Roberto Giambruno, Francesco Nicassio

**Affiliations:** ^1^ Center for Genomic Science of IIT@SEMM, Fondazione Istituto Italiano di Tecnologia, Milano, Italy; ^2^ Institute of Biomedical Technologies, National Research Council, Segrate, Italy

**Keywords:** protein-RNA interactions, proximity biotinylation, APEX2, affinity purification, affinity purification coupled to mass spectrometry, APEX-seq, dCas13

## Abstract

Proximity ligation technologies are extremely powerful tools for unveiling RNA-protein interactions occurring at different stages in living cells. These approaches mainly rely on the inducible activity of enzymes (biotin ligases or peroxidases) that promiscuously biotinylate macromolecules within a 20 nm range. These enzymes can be either fused to an RNA binding protein or tethered to any RNA of interest and expressed in living cells to biotinylate the amino acids and nucleic acids of binding partners in proximity. The biotinylated molecules can then be easily affinity purified under denaturing conditions and analyzed by mass spectrometry or next generation sequencing. These approaches have been widely used in recent years, providing a potent instrument to map the molecular interactions of specific RNA-binding proteins as well as RNA transcripts occurring in mammalian cells. In addition, they permit the identification of transient interactions as well as interactions among low expressed molecules that are often missed by standard affinity purification strategies. This review will provide a brief overview of the currently available proximity ligation methods, highlighting both their strengths and shortcomings. Furthermore, it will bring further insights to the way these technologies could be further used to characterize post-transcriptional modifications that are known to regulate RNA-protein interactions.

## Introduction

RNA and proteins are constantly interacting in living cells at multiple stages and their interaction is fundamental for their biological functions (Yeo, 2014; Hentze et al., 2018). RNA molecules can transiently interact with RNA binding proteins (RBPs) or lying in complexes with proteins, forming ribonucleoprotein complexes. For RNAs, the interaction with proteins is required for their biogenesis and functions, regulating several aspects of cell biology including transcription, splicing and translation. For proteins, interaction with RNAs is necessary to maintain the folding or the integrity of a multi-subunit complex, to direct the catalytic moiety to specific targets or compartments, and to modulate the protein biological activity. Physical interaction is mediated by RNA-binding domains and intrinsically disordered regions of RBPs, which can recognize specific RNA structures (hairpins, stems, or loops), RNA sequence motifs or simply have a high affinity to bind RNA molecules. Importantly, RNA-protein interactions are not usually stable and are frequently regulated by post-transcriptional and post-translational events that modulate the binding affinity (Lewis et al., 2017; Spadotto et al., 2020). Since RNA-protein interactions play a key role in molecular, cellular and developmental biology it is not surprising that alterations can affect cellular homeostasis and have been linked to many human diseases, including neurodegenerative disorders and cancer (Gebauer et al., 2021; Kelaini et al., 2021).

Several methods have been developed to characterize RNA-protein interactions at global level, distinguished into RNA-centric or protein-centric approaches. Usually, the RNA or protein of interest is isolated by affinity purification from cells and binding partners are identified by high-throughput mass spectrometry and RNA sequencing approaches, respectively (McHugh et al., 2014; Giambruno et al., 2018; Gerber, 2021). Despite being powerful, these methodologies often miss interactions that are transient or involve low abundant molecules. The use of crosslinking agents can sensibly increase the number of detected interactions, although introducing biases towards pyrimidine-reach RNA regions as well as increasing the risk of false positives and non-direct interactions (Ramanathan et al., 2019). An alternative strategy is provided by Proximity Dependent Biotinylation (PDB) approaches, in which bacterial enzymes are used to biotinylate functional groups in proximity of the protein of interest (<20 nm) and used to detect protein-protein and protein-nucleic acid interactions (Ramanathan et al., 2018; Fazal et al., 2019; Padrón et al., 2019). The biotinylated molecules are isolated through biotin-streptavidin interaction, which is efficient and specific even under denaturing conditions, a condition which allows the enrichment of true interactors and removal of non-specific binders. The main advantages are: 1) the biotinylation occurs in living cells, preserving the biological cellular environment and avoiding any artificial interactions that might occur during cell lysis; 2) it tracks stable as well transient interactions, even at picomolar scale, without the need of any crosslinking step; 3) it is compatible with OMICS technologies allowing the global identification of the interactions occurring between the RNA or protein of interest in cells.

The use of PDB approaches to assess protein-protein interactions, protein cellular localization and compartmentalization, including the regulation mediated by post-translational modifications have been recently reviewed (Dionne and Gingras, 2022). The focus of this mini review is exclusively related to the application of these approaches to unveil RNA-protein interactions and their dynamics in living cells, avoiding the use of crosslinking agents to stabilize molecular interactions.

## PDB enzymes

Biotin ligases are enzymes able to convert, in the presence of ATP, biotin into an active biotin-5-AMP intermediate that is covalently linked to primary amines (epsilon group of lysine residues and protein N-termini) of proximal proteins. The first used biotin ligase is the *E. Coli* Bifunctional ligase/repressor enzyme carrying the R118G mutation (BirA*) that promiscuously biotinylates any proximal protein (Roux et al., 2012). Several enzymes have been purified from different bacteria and engineered to enhance their catalytic activity towards protein substrates. Among them, the BASU protein derived from *Bacillus subtilis* that biotinylates protein within 30 min (Ramanathan et al., 2018); and the TurboID protein which is a mutated form of BirA* able to biotinylate proteins in less than 10 min (Branon et al., 2018).

Alternatively, the activity of the mutated form of the heme ascorbate peroxidase enzyme APEX2 (Apurinic/Apyrimidinic Endodeoxyribonuclease 2), has been exploited for PDB approaches. APEX2 is the more active variant of the initial APEX enzyme derived from soybean (Lam et al., 2015). Upon hydrogen peroxide (H_2_O_2_) treatment, APEX2 converts phenol substrates into short-lived phenoxyl radicals with a half-life of less than one millisecond that covalently attach electron-rich amino acids and nucleotides with a special preference for tyrosine and guanine, respectively (Qin et al., 2021a).

The biotinylation process mediated by PDB enzymes is strongly dependent on the abundance, length, composition and structure of the targeted molecules. Moreover, substrate regions have to be exposed and freely available to the labeling of the PDB enzyme. Hence, the labeling intensity is not directly correlated with the strength of the interaction (Mair and Bergmann, 2022). A detailed summary of the various PDB enzymes currently used in molecular biology and their mechanism of actions can be found elsewhere (Samavarchi-Tehrani et al., 2020; Qin et al., 2021b).

## RNA centric methods

RNA centric PDB methods assess which proteins are interacting with a selected RNA transcript, in living cells. The PDB enzyme is recruited to the RNA of interest and, once activated, it starts biotinylating any protein present in proximity over time. Even proteins that transiently interact with the targeted RNA transcript can be covalently labeled with one or multiple biotin molecules. The resulting biotinylated proteins are then affinity purified under denaturing conditions and identified by MS-based proteomics through either label free quantification or stable isotope labeling methods, as previously summarized (Lindemann et al., 2017; Giambruno et al., 2018). Differently from standard RNA centric methods, RNA centric PDB strategies are highly sensitive especially for the detection of transient interactions and do not require any step to preserve RNA-protein interactions prior cell harvesting. Thus, these methods maximize the sensitivity without increasing false positive interactions (Ramanathan et al., 2019). However, they cannot provide information about: 1) whether the protein is a direct RNA-binder and to which portion of the RNA is bound; 2) if the identified proteins simultaneously bind the RNA or at different stages; 3) whether the identified interactors belong to multiprotein complexes; 4) if the detected RNA-protein interaction is mediated by RNA post-transcriptional modifications.

**FIGURE 1:**
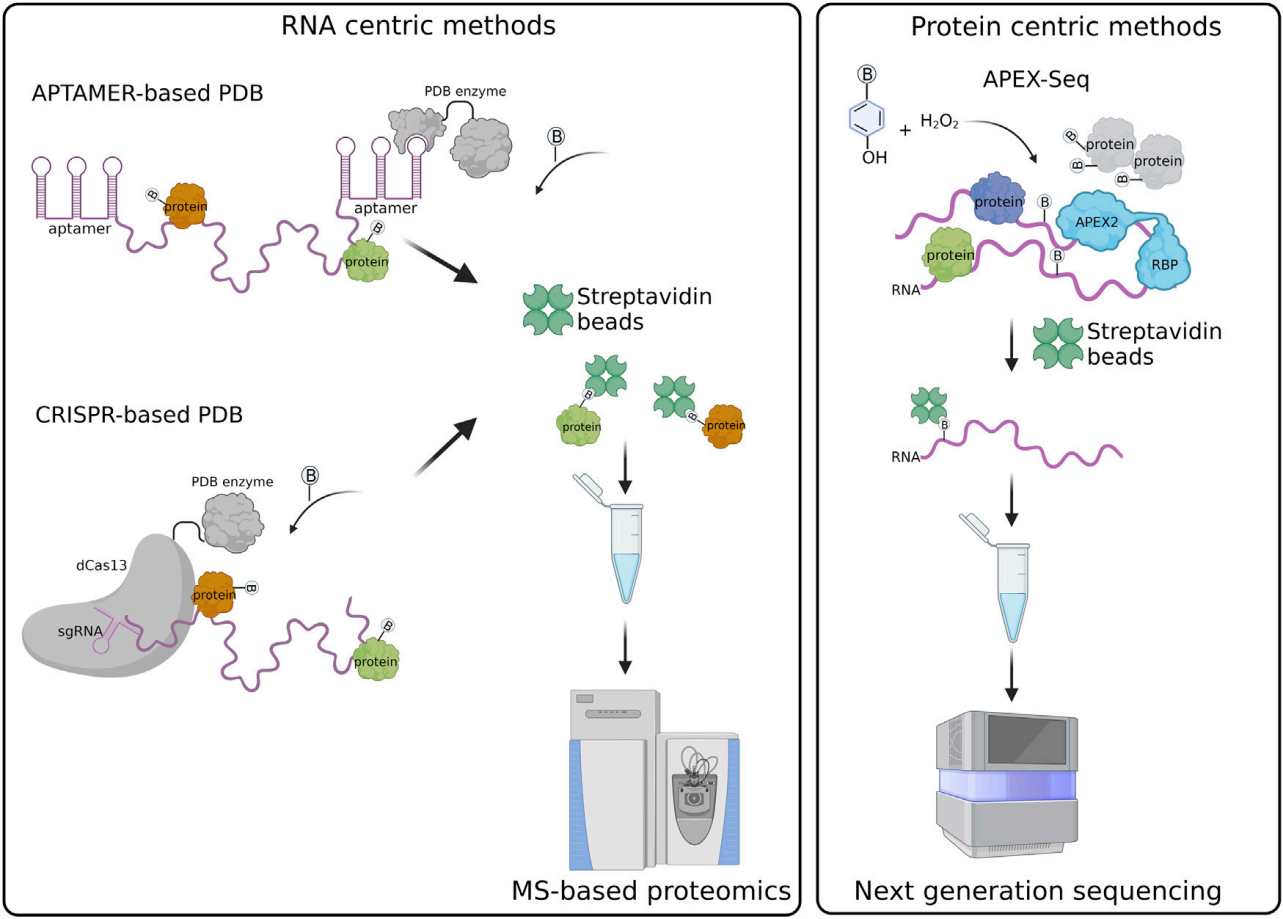
Schematic representation of RNA-centric and protein centric PDB created with BioRender.com.

Currently, the main approaches are based on 1) Aptamer; 2) Clustered Regularly Interspaced Short Palindromic Repeats (CRISPR). They are represented in Figure 1.

*Aptamer-based PDB* exploits the tagging of the RNA of interest with the MS2 or BoxB aptamers that are specifically recognized and bound by the MS2 coat protein (MCP) and λN peptide fused in frame with the labeling enzyme, respectively (Weissinger et al., 2021). An example is represented by the RNA-protein interaction detection—mass spectrometry (RaPID-MS) strategy (Ramanathan et al., 2018), where the RNA of interest is expressed in living cells tagged with three BoxB aptamers located both at the 5′ and 3′ ends. The aptamers are bound by the co-expressed enzyme BASU carrying at its N-terminus the λN-peptide that allows the tethering of the PDB enzyme to the BoxB-tagged RNA. BASU activity is then promoted by the administration of exogenous biotin in the cell culture medium. Biotinylated proteins are affinity purified under denaturing conditions and analyzed by liquid chromatography coupled to tandem mass spectrometry (LC-MS/MS). In this approach the RNA transcripts are overexpressed, therefore it is better suitable for the identification of proteins bound to specific RNA motifs or to compare the interactome of a wild-type versus mutated RNA sequences (Ramanathan et al., 2018). It can be also exploited to identify the host-protein interactions of exogenous transcripts, such as viral RNA transcripts, which are usually expressed at high levels in infected cells, as reported for the Zika and SARS-CoV-2 viruses (Ramanathan et al., 2018; Giambruno et al., 2022).

An alternative strategy is RNA-BioID (Mukherjee et al., 2019) that has been used to analyze the protein interactome of the endogenous *β*-Actin RNA through the insertion of 24 repeats of the MS2 aptamer at the 3′UTR of the gene. The MS2-tagged RNA is bound by a stably expressed MCP-BirA*, which biotinylates proteins associated with the RNA. The labeled proteins are then purified and identified by LC-MS/MS. However, the BirA* proximity labeling time was conducted for 24 h, a relatively long time during which multiple RBPs can enter in proximity with the targeted RNA, therefore being biotinylated. Thus, strongly reducing the signal-to-noise ratio of the true interactors identified by this strategy.

The time for the biotinylation has been sensibly minimized through the development of the MS2-based APEX method (Han et al., 2020). The authors co-expressed in living cells the MCP-APEX2 enzyme and the human telomerase RNA (hTR) carrying a tag of 4x MS2 repeats fused to its 5′ RNA. The main advantage is represented by the fact that APEX2 has an extremely fast kinetic promoting proximal protein biotinylation in less than a minute. Thus, APEX2 allows the detection of transient and dynamic RNA-protein interactions to the same extent of those interactions that are more stable and, hence, can be detected more easily by standard biochemical approaches. In addition, the shorter number of aptamer repeats as compared to the one used in RNA-BioID better preserves the biological properties of the tagged RNA (i.e., MS2-tagged hTR) (Laprade et al., 2020). However, this strategy works well only for overexpressed RNAs (Han et al., 2020).

*CRISPR PDB* approach exploits the activity of the dCas13 enzyme, which specifically binds RNA sequences under the guidance of single guide RNAs (sgRNAs), without cleaving the RNA or targeting DNA sequences (Abudayyeh et al., 2017). The PDB enzyme is fused in frame to the dCas13 and therefore recruited to the endogenous RNA target.

Four similar CRISPR PDB tools have been developed: 1) CARPID (CRISPR assisted RNA-protein interaction detection method) (Yi et al., 2020), 2) Cas13-based APEX method (Han et al., 2020), 3) CBRPP (CRISPR-based RNA proximity proteomics) (Li et al., 2021), and 4) RPL (RNA proximity labeling) (Lin et al., 2021). They have in common the use of a fusion protein composed of catalytically inactive Cas13 variants (dCas13 or dRfxCas13d) and a PDB enzyme (APEX2, BASU and BioID2) (Han et al., 2020; Yi et al., 2020; Lin et al., 2021). Differently from aptamer-based strategies, CRISPR PDB approaches directly target endogenous RNA transcripts in living cells, without the need of a pre-labelling step. The fusion protein dCas13-PDB enzyme is tethered to the RNA of interest by a single or multiple sgRNAs. The number of sgRNAs is chosen according to the length of the targeted RNA. In the case of lncRNA, such as XIST and MALAT1, a set of different sgRNAs has been used to probe the different regions of the RNA (Yi et al., 2020). As the secondary structures of the targeted RNA can influence sgRNA pairing, multiple sgRNAs should be tested to select those that are effective (Han et al., 2020). The recruitment of the dCas13-APEX2 to the RNA target can be improved by different strategies, such as: 1) the insertion of a double strand RNA binding domain (dsRBD) at the C-terminus of the fusion protein, which stabilize the protein-RNA complex (Han et al., 2020); 2) the adoption of inducible expression systems that regulate the expression of the dCas13-APEX2 in cells and enhance the signal-to-noise labeling ratio (Han et al., 2020; Li et al., 2021); 3) the addition of a nuclear export sequence (NES) or nuclear localization signal (NLS) to concentrate the fusion protein in the same cellular compartment of the targeted RNA (Lin et al., 2021).

RNA centric PDB methods require the use of appropriate experimental controls. It is advisable to include in the analysis an unrelated RNA, with length and GC-content similar to the RNA of interest, whose results can be used to measure the experimental background. Moreover, an RNA with known interacting partners can be used as a positive control, assessing the efficacy of the strategy and the sensitivity of the assay and the related instrumentation (Table 1).

**TABLE 1:** Summary of strengths, limitations and available tools of the current PDB strategies.

	RaPID-MS	RNA BioID	MS2-based APEX method	CRISPR proximity biotinylation tools	Purification of protein/RNA complexes	APEX-Seq
BAIT	• BoxB-tagged RNA	• MS2-tagged RNA	• MS2-tagged RNA	• Endogenous RNA	• APEX- tagged protein	• APEX- tagged protein
PREYS	• Endogenous proteins	• Endogenous proteins	• Endogenous proteins	• Endogenous proteins	• Endogenous RNAs	• Endogenous RNAs
PURIFICATION APPROACH	• Streptavidin pull-down of biotinylated proteins	• Streptavidin pull-down of biotinylated proteins	• Streptavidin pull-down of biotinylated proteins	• Streptavidin pull-down of biotinylated proteins	• Streptavidin pull-down of biotinylated proteins	• Streptavidin pull-down of biotinylated RNAs
STRENGHTS AND LIMITATIONS	✓ No crosslinking agents	✓ No crosslinking agents	✓ No crosslinking agents	✓ No crosslinking agents	✓ No crosslinking agents	✓ No crosslinking agents
	✓ Low amount of material	* High amount of material	✓ Low amount of material	* High amount of material	✓ Low amount of material	✓ Low amount of material
	✓ Fast	* Slow	✓ Fast	* Slow	✓ Fast	✓ Fast
	✓ Not expensive	* Expensive	✓ Not expensive	* Expensive	✓ Not expensive	* Expensive
	✓ Easy to use technology	* Laborious technology	✓ Easy to use technology	* Laborious technology	✓ Easy to use technology	* Laborious technology
	* Not applicable to endogenous RNAs	✓ Applicable to endogenous RNAs	* Not applicable to endogenous RNAs	✓ Applicable to endogenous RNAs	* Not applicable to endogenous proteins	* Not applicable to endogenous proteins
	* Presence of not direct interactors	* Presence of not direct interactors	* Presence of not direct interactors	* Presence of not direct interactors	* The identified RNAs cannot be address to a single RBP but rather to the whole protein complex	* Presence of not direct interactors
	* The tag can alter the localization and interaction profile of the bait	* The tag can alter the localization and interaction profile of the bait	* The tag can alter the localization and interaction profile of the bait	* The off-target binding of the sgRNAs and the presence of the dCas13 can alter the interaction profile of the bait	* The tag can alter the localization and interaction profile of the bait	* The tag can alter the localization and interaction profile of the bait
AVAILABLE TOOLS	• Plasmid to clone BoxB‐tagged RNAs (Addgene #107253)		• Plasmid encoding for MCP-APEX2 (Addgene #154936)		• Plasmid encoding for CARPID BASU-dCasRx (Addgene #153209), CARPID dCasRx-BASU (Addgene #153303), dCas13d-dsRBD-APEX2 (Addgene #154939)	• Addgene plasmids encoding for APEX2-OMM (#79056), ERM-APEX2 (#79055), mito-APEX2 (#72480), APEX2-SENP (#129276), APEX2-eIF41 (#129645), APEX2-eIFE1 (#129644), C1-APEX2 (#129641), APEX2-FBL (#187577), APEX2-SRSF7 (#187582), APEX2-SRSF1 (#187575), APEX2-PML (#187583), APEX2-SP100 (#187584), APEX2-NPAT (#187585), APEX2-LMNA (#187576)
	• plasmid for mammalian expression of lN‐HA‐BASU (Addgene #107250)					
	• Plasmid expressing the positive control BoxB-EDEN15 (Addgene #107252					
PROPER CONTROLS	• Scramble RNA of the same length and similar GC-content	• Scramble RNA of the same length and similar GC-content	Scramble RNA of the same length and similar GC-content	• Non targeting sgRNAs	• APEX2-GFP	• APEX2-GFP
	• Positive control RNA with known interactors	• Positive control RNA with known interactors	• Positive control RNA with known interactors	• Positive control RNA with known interactors	• APEX2-tagged protein located on a different cellular compartment	• APEX2-tagged protein located on a different cellular compartment
References (DOI)	- 10.1038/nmeth.4601	- 10.1073/pnas.1820737116	- 10.1073/pnas.2006617117	- 10.1073/pnas.2006617117	- 10.1093/nar/gkaa376	- 10.1016/j.cell.2019.05.027
				- 10.1038/s41592-020-0866-0		
				- 10.1080/15476286.2021.1873620	- 10.1083/jcb.202002129	- 10.1016/j.molcel.2019.07.030
				- 10.1080/15476286.2021.1917215		

## Protein centric methods

Protein centric PDB methods allow the characterization of the RNA transcripts that are bound to or in proximity of a protein of interest through next generation sequencing (NGS) approaches. Differently from standard protein centric methods, this strategy does not require the use of an antibody for the protein target and, hence, can be applied to any protein or protein isoform (Qin et al., 2021b). The protein bait is expressed in living cells as a fusion protein having either at its N- or C-terminus a spacer containing a tag used for detection (i.e. FLAG/HA epitope) and followed by a PDB enzyme. So far, researchers have preferred the use of APEX enzymes, which guarantee a faster labeling time compared to biotin ligase enzymes (Bosch et al., 2021). Once expressed and activated by the administration of phenol-biotin into the cell culture medium followed by H_2_O_2_ treatment, the APEX enzyme starts biotinylating any macromolecule present in its proximity. The cells are then harvested and biotinylated proteins, together with their associated RNA transcripts, pulled down through streptavidin beads. The RNA is extracted from the beads, purified and analyzed by standard NGS. This strategy has been extremely helpful for the characterization of the RNA transcripts associated with subcellular compartments, such as stress granules (SGs) and the nuclear lamina, using protein markers such G3BP1 and LAMIN B1, as baits (Somasekharan et al., 2020; Tran et al., 2021).

In the APEX-Seq approach, APEX2 is used to directly biotinylate nucleic acids (Figure 1). Thus, biotinylated RNA transcripts are affinity purified through streptavidin beads and analyzed by NGS (Fazal et al., 2019; Padrón et al., 2019; Zhou et al., 2019). APEX-Seq can be used to efficiently map both the proteins and RNAs interacting with a protein of interest, used as bait. This approach has proven extremely useful for the definition of protein-RNA and protein-protein interactions occurring within cellular structures or membrane-less organelles without the need of isolation approaches (Fazal et al., 2019; Padrón et al., 2019). APEX-Seq was applied for the characterization of the RNA-protein interaction patterns of different subcellular organelles and compartments including the inner (Mito-APEX2) (Fazal et al., 2019; Zhou et al., 2019) and outer mitochondrial membrane (APEX2-OMM) (Fazal et al., 2019), the endoplasmic reticulum membrane [ERM-APEX2 or C1(1-29)-APEX2] (Fazal et al., 2019; Padrón et al., 2019) and the nuclear pore (APEX2-SENP) (Fazal et al., 2019). Furthermore, APEX-Seq was employed to studying RNA-protein interactions in macromolecular complexes, such as SGs (APEX2-eIF4A1) or the translation initiation complex (APEX2-eIF4A1 and APEX2-eIF4E1) (Padrón et al., 2019). This approach has been exploited also to analyze the dynamics of RNA-protein interactions upon perturbations, as shown by the analysis of the pattern of RNAs recruited by eIF4A1 to SGs in response to different types of stress (Padrón et al., 2019) and the analysis of RNA interaction patterns of the outer mitochondrial membrane in response to drug perturbations (Fazal et al., 2019).

Recently, APEX-Seq has been used to investigate even membraneless domains, using as bait their specific markers. Relevant examples are APEX2-FBL for the nucleolus; APEX2-SRSF7, APEX2-SRSF1, and APEX2-RNPS1 for nuclear speckles; APEX2-SMN2 for Cajal bodies; APEX2-SAM68 for the SAM68 bodies; APEX2-PML and APEX2-SP100 for the PML bodies; APEX2-NPAT for the histone locus bodies; and APEX2-LMNA for the nuclear lamina (Barutcu et al., 2022).

The labeling of RNA transcripts can be improved by using biotin-aniline as peroxidase substrate. Conversely to proteins, APEX2 biotinylates RNA transcripts approximately 3-fold times more in the presence of biotin-aniline compared to biotin-phenol (Zhou et al., 2019). Nevertheless, APEX2 can also biotinylate DNA hence, it is necessary to remove any traces of DNA before analyzing biotinylated RNA transcripts (Matěju and Chao, 2022).

To correctly define the RNA interactome of a given bait, it is preferable to conduct the same purification strategy on multiple baits that preferably localize to different cellular compartments. This can help to properly assess the experimental background and identify those frequently recurring RNA-protein interactions.

## Discussion

The characterization of RNA-protein interactions in living cells has to take into consideration four aspects: 1) the bait has to be soluble to be affinity purified; 2) the preys must interact with the bait for a sufficient time and amount to be detected; 3) RNA-protein interactions have to be preserved during the whole affinity purification procedure; 4) the signal to noise ratio must be high to permit the correct identification of true interactors and minimize number of false positives. To this extent, PDB approaches have sensibly favored the biochemical workflow applied to assess the interactome of an RNA or a protein of interest. The biotinylation process occurs in living cells where the cellular environment is preserved. This eliminates the formation of false positive RNA-protein interactions that can occur during cell lysis, when the cellular membranes are disrupted. At the same time, any bait can be purified thanks to the possibility of adopting even denaturing cell lysis buffers.

The strong affinity between streptavidin and biotin (Kd 10^−14^ M) (Michael Green, 1990) allows purification of biotinylated molecules under denaturing conditions, including the presence of high salt and detergents in the purification buffer. Thus, only biotinylated molecules are purified for the subsequent identification. However, PDB methods cannot discriminate between direct and proximally located interactors of a given bait. In addition, many RBPs have a promiscuous association with RNA transcripts, especially when they are abundantly expressed (Nielsen et al., 2016; Protter et al., 2018; Corley et al., 2020). Therefore, if a protein is directly and specifically interacting with an RNA, it should be verified by orthogonal techniques.

PDB technologies rely on the activity of a bacterial enzyme fused or tethered to the molecule of interest and, hence, poses important limitations. The enzyme may alter the biological properties of the targeted molecules. For instance, the paraspeckle proteins NONO, PSPC1, and EWSR1 tagged with APEX2 at their N-terminus showed a non-physiological localization compared to the respective endogenous proteins (Barutcu et al., 2022). Thus, it is necessary to assess that the tagged molecule maintains its proper localization and, if possible, biological function. In addition, the bacterial enzyme can interfere with the binding of protein or cellular RNAs to one or multiple portions of the tagged bait. The dCas13 protein, for instance, is a 130 KDa protein that once tethered to the targeted RNA can sterically outcompete RBPs that transiently bind to the RNA regions where the dCas13 is present (Han et al., 2020). The same issue may occur when fusing a PDB enzyme to a protein of interest, thus impacting its interactions (Qin et al., 2021a).

One of the current limits for the characterization of RNA-protein interactions is the difficulties in performing an unbiased identification and characterization of the post-transcriptional modifications present in the RNA transcripts that are interacting with cellular proteins. RNA post-transcriptional events have been recognized with important regulatory functions and are known to regulate RNA folding into secondary structures and the propensity of the RNA to interact with partners (Li and Mason, 2014). Protein centric PDB technologies may potentially be exploited to detect post-transcriptional modifications that decorate RNA transcripts while interacting with proteins. However, the current NGS protocols require the conversion of RNA into cDNA prior to sequencing. Thus, eliminating any information related to the presence of post-transcriptionally modified nucleotides. Nanopore direct RNA-Sequencing (DRS) has emerged as a new technology that offers for the first time the possibility to sequence full-length native RNA molecules, allowing the study of RNA modifications in an unbiased way and at single nucleotide resolution. In the next future, the coupling of nanopore DRS protocols downstream of protein centric PDB technologies will allow for a protein of interest the identification of the associated RNAs and their post-transcriptional modifications.
